# Factors associated with COVID-19 vaccine uptake and hesitancy among women of reproductive age in Mozambique

**DOI:** 10.3389/fpubh.2025.1484477

**Published:** 2026-01-12

**Authors:** Lauren Gilliss, Cremildo Manhiça, Helen Kuo, Azarias Mulungo, Akum Aveika, Nordino Machava, Fred Van Dyk, Charfuddin Sacoor, Celso Monjane, Ivalda Macicame, Agbessi Amouzou, Almamy Malick Kanté

**Affiliations:** 1 Johns Hopkins Bloomberg School of Public Health, Baltimore, MD, United States; 2 JSI Research & Training Institute, Inc., Arlington, VA, United States; 3 Instituto Nacional de Saude, Maputo, Mozambique; 4 Centro de Investigação em Saude de Manhiça (CISM), Maputo, Mozambique

**Keywords:** COVID-19, determinants of vaccine hesitancy, vaccine uptake, mobile phone surveys, Mozambique

## Abstract

**Objective:**

Vaccine hesitancy has become one of the biggest challenges in combating the COVID-19 pandemic globally. This paper aims to determine the factors associated with COVID-19 vaccination and hesitancy among women of reproductive age in Mozambique.

**Methods:**

A cross-sectional mobile phone survey was conducted among women ages 15–49 to test the use of mobile phone interviews to collect data on household deaths and other topics related to pregnancy, delivery care, women’s empowerment, and COVID-19 vaccination. We calculated COVID-19 vaccination coverage rates, defined as women who received at least one dose of the COVID-19 vaccine, and described reasons for not taking the vaccine. Multivariate logistic regression was used to assess factors associated with COVID-19 vaccine uptake in the study population. All estimates were adjusted using post-adjustment weighting based on the raking approach to redress the sample to be nationally representative.

**Results:**

The mobile phone survey response rate was estimated at 39.1% (*n* = 13,235). The adjusted COVID-19 vaccination rate was estimated at 77.2% [95%CI: 74.9–79.5] among women aged 15–49. Among the unvaccinated women, about 11.9% were hesitant to take the COVID-19 vaccine if offered. The primary reasons reported for not taking the vaccine were the dislike of needles (17.1%), COVID-19 vaccine safety concerns (12.0%), COVID-19 vaccine effectiveness concerns (10.2%), and medical reasons (5.6%). We found a positive and significant association between COVID-19 vaccine acceptance and age group, education, marital status, province and place of residence, and women who used maternal health facility services. However, women empowerment factors were not significantly associated with increased COVID-19 vaccine acceptance.

**Conclusion:**

Our findings showed high rates of COVID-19 vaccination among women aged 15–49 with social, economic, and residential inequalities. To increase COVID-19 vaccine coverage among women in Mozambique, more effort should be put into vaccinating younger women, uneducated women, and those delivering outside a health facility. More attention should also be given to factors related to vaccine acceptance and hesitancy in Mozambique.

## Introduction

The coronavirus SARS-CoV-2 (COVID-19), has drastically impacted countries globally. Its spread and severity have been influenced by factors such as resource allocation, availability of tests, strength of existing health systems, effective risk communication, adherence to recommended preventive measures, and vaccine acceptance or hesitancy (1–4). Low-and middle-income countries (LMICs) have faced economic challenges, increased pressure on health systems and difficulties in securing and distributing COVID-19 vaccines (5). As vaccination remains a critical strategy for reducing severe illness and death, and for controlling the pandemic, many studies have examined vaccine access and uptake across various populations (1, 6–10). However, it is essential to consider the country-specific context when analyzing the factors contributing to vaccine acceptance and hesitancy. In Mozambique, there is limited research on the predictors of the COVID-19 vaccination uptake and the factors influencing individuals’ willingness to be vaccinated.

Mozambique’s first case of COVID-19 was confirmed on March 22, 2020, and the President of Mozambique declared a state of emergency in response to the pandemic on April 20, 2020 (11). The situation escalated to a state of public calamity on September 7, 2020 (11). The Government of Mozambique announced its vaccination strategy on March 5, 2021, which included a target of vaccinating 16.8 million Mozambicans (11). As of June 2023, about 34.9 million doses of COVID-19 vaccines had been administered in Mozambique, with about 63% of the population being fully vaccinated. About 233,417 confirmed COVID-19 cases and 2,243 deaths due to COVID-19 had been reported in Mozambique (12).

Vaccine acceptance is commonly discussed throughout the literature relating to vaccine uptake (1, 2, 6, 8, 13–16). Acceptance rates can vary based on trust in vaccines and the institutions administering them, and the spread of misinformation (5). To reduce the public health burden of COVID-19, acceptance must be translated into uptake- a complex process influenced by factors such as access, health system capacity, and infrastructure (5, 13).

Studies have documented a wide range of COVID-19 vaccine acceptance across and within sub-Saharan African countries, with low rate of 7% in a 2021 study in southeast Nigeria and high rate of 98% in a 2021 phone survey study in Ethiopia (1, 15, 17–19). A 2021 narrative review further found vaccine acceptance rates ranging from 50% in Zimbabwe to 92% in Ethiopia (18). Commonly reasons given for COVID-19 vaccine acceptance included personal and family protection against COVID-19 infection (20).

Vaccine hesitancy has emerged as a major global challenge in efforts to control the COVID-19 pandemic (1, 10, 18). The World Health Organization (WHO) defines vaccine hesitancy as a “delay in acceptance or refusal of vaccination despite availability of vaccination services” (21). Individuals who are vaccine-hesitant may delay vaccination, accept some vaccines but not others, or refuse vaccines altogether (1, 2). Research consistently shows that women are more likely to be vaccine-hesitant than men (1, 7, 22–25). Common reasons for COVID-19 vaccine hesitancy included concerns about the vaccine’s novelty and safety, fear of potential side effects, and general vaccine avoidance (1, 2, 10). In sub-Saharan Africa, mistrust of authorities involved in COVID-19 vaccine development and distribution – such as vaccine manufacturers and pharmaceutical companies- has also contributed to vaccine hesitancy (1, 16, 24).

In Mozambique, a study conducted in March 2021, before the availability of COVID-19 vaccines, reported that 65% of the general population and 87% of healthcare workers surveyed expressed willingness to take the COVID-19 vaccine if it was available (8). Another study, conducted in Mozambique between September 2020 and March 2021, reported fluctuations in vaccine acceptance over time, with acceptance significantly associated with levels of institutional trust (26). Since then, the COVID-19 situation in Mozambique has evolved with vaccines becoming more widely available, which could affect perceived vaccine acceptability and factors related to uptake. Understanding these factors is critical to inform strategies to improve vaccine coverage and address gender-specific barriers. This study aims to determine the COVID-19 vaccine uptake, hesitancy, and associated factors among women of reproductive age in Mozambique.

## Methods

### Study design

A cross-sectional mobile phone survey was conducted in Mozambique among women ages 15–49 to test the use of mobile phone interviews to collect data on child and adult deaths and other topics related to pregnancy, delivery care, women’s empowerment, and COVID-19 vaccination status (27). The Rapid Mortality Mobile Phone Surveys (RaMMPS) project in Mozambique aimed to test rapid approaches for measuring population-based child mortality, maternal health coverage, and COVID-19 related statistics during a health crisis to understand its impact on decision-making related to seeking healthcare, while avoiding face-to-face interviews (28).

### Data collection

This study reports data collected by the RaMMPS study that used a subsample from an existing national sample registration system known as the Countrywide Mortality Surveillance for Action (COMSA) in Mozambique (29). The COMSA study maintained a list of households that provided phone numbers. A province-stratified random sample of 48,271 households with phone numbers was selected for the survey to reach 15,000 women of reproductive age. The RaMMPS sample identified eligible women of reproductive age within the selected households. Data collectors were evaluated for proficiency in Portuguese and local languages. Selected candidates received ten days of training on protocols, ethics, best practices, and use of the Open Data Kit (ODK) (30), including pilot testing. Thirty-two interviewers and four supervisors (89% women) were recruited. A call center was established to support interviewer–respondent communication.

The RaMMPS adapted Demographic Health Survey (DHS) tools (31), including a pregnancy history module to assess child mortality. Additional modules covered: (1) maternal health care seeking, including ANC, facility delivery and birth assistance questions, (2) women’s empowerment, such as mobile phone ownership and use, and participation in family healthcare-related decision making; (3) household composition and deaths since January 2020, including identification of possible COVID-19 deaths using Vital Strategies and WHO tools (32); and (4) COVID-19 vaccine acceptance and hesitancy using The World Bank tools (33) and Quinn and colleagues measures (34). The vaccine module captured the number and type (name of the product) of COVID-19 vaccine doses received, willingness to get vaccinated if available among unvaccinated respondents, and reasons for vaccine refusal.

Computer-assisted telephone interviews (CATI) were used, and the data collection was conducted between March–July 2022; and 13,235 completed interviews with women aged of 15–49 were included in the study (27).

### Data analysis

RaMMPS relied on mobile phone surveys limiting participation to individuals with mobile phones access. To address potential biases from phone ownership (coverage) and survey non-response (35), we employed post-stratification techniques. The 2017 Mozambique Census population data (36) was used to derive the distribution of women aged 15–49 by age, education, province and place of residence, and household size, which was applied in the raking approach to adjust the final sampling weights (37), using the STATA package “*svycal*” (38).

In this study, COVID-19 vaccine acceptance is defined as “having received at least one dose of a COVID-19 vaccine and, if not, willingness to take the COVID-19 vaccine when it is available” (39). The dependent variable was whether a woman aged 15–49 reported having received at least one dose of the COVID-19 vaccine. The proportion of unvaccinated women who reported that they would take the vaccine if it was available and offered to them was determined. Women who were unlikely, undecided, or definitely not planning to take the COVID-19 vaccine were asked to report the reasons for their hesitancy.

Descriptive statistics were presented as percentages. The proportion of vaccinated women was compared by demographic and residential characteristics using chi-square tests. We conducted logistic multivariate analyses to investigate factors associated with COVID-19 vaccination. We produced weighed estimates (percentages and odds ratios) with 95 percent confidence intervals (CI). All statistical analyses were conducted using STATA statistical package version 17 (40).

## Results

### Participants’ characteristics

The mobile phone survey response rate was estimated at 39.1% (*n* = 13,235). Most respondents were in the 20–29 age group (37.7%), attended primary level of education (49.8%), and were married or cohabiting with their partner (74.7%). The participants represented all eleven of Mozambique’s provinces, and nearly two-thirds resided in rural areas (61.7%). Almost half (46.0%) of respondents reported owning the mobile phone they were using for the interview, and 68.6% reported having access to the mobile phone whenever they wanted to use it. Eight out of ten (83.3%) women had ever given birth. Among those who had given birth in the past 24 months prior to the survey, almost all (98.6%) reported having at least one antenatal care (ANC) visit, but only 23.4% achieved four or more visits. About 86% of women delivered at a health facility. More than one-third (37.5%) of the respondents usually make decisions about their health care, and 47.0% reported that they usually make decisions for child healthcare (Table 1).

**Table 1:** Socio-demographic characteristics of respondents.

Characteristics	Categories	Variables	Unweighted distribution (%)	Weighted distribution (%)	Weighted count (*n*)
Demographics	Age group	Less than 20	5.2	22.9	3,030
		20–29	35.1	37.7	4,996
		30–39	32.1	23.1	3,061
		40–49	27.5	16.2	2,148
	Education	None	10.7	22.4	2,970
		primary	37.0	49.8	6,587
		Secondary and more	52.3	27.8	3,679
	Marital status	Married/cohabited	69.1	74.7	9,881
		Widowed/Divorced/Separated	13.7	9.4	1,245
		Never being married	17.2	15.9	2,108
Birth history	Never gave birth	Yes	87.9	83.3	11,022
		No	12.1	16.7	2,213
	Number of birth (*n* = 11,022)	One	20.9	26.0	2,861
		2_4	57.6	52.1	5,738
		5+	21.4	22.0	2,423
	Currently pregnant (*n* = 11,022)	Yes	10.4	15.3	1,688
		No	89.6	84.7	9,333
	Antenatal care (ANC) services (*n* = 4,604)	Yes	99.2	98.6	4,539
		No	0.8	1.4	65
		Less than 4	84.6	76.6	3,479
		4 ANC or more	15.4	23.4	1,060
	Place of delivery (*n* = 4,604)	Health facility	91.7	14.3	657
		Home	8.3	85.7	3,947
Empowerment (decision making)	Phone ownership	Respondent	63.6	46.0	6,084
		Husband/conjoint	22.5	37.7	4,993
		Other	14.0	16.3	2,158
	Need permission to use phone (*n* = 6,085)	Yes	8.9	14.6	888
		No	91.1	85.4	5,197
	Can access to phone if needed	Yes	82.8	68.6	9,081
		No	17.2	31.4	4,154
	Taking decisions on her own health	Respondent	51.4	37.5	4,963
		Husband/conjoint	33.9	45.0	5,955
		Other	14.7	17.5	2,317
	Taking decisions on her children health	Respondent	52.5	47.0	6,216
		Husband/conjoint	42.3	47.6	6,300
		Other	5.2	5.4	720
Place of residence	Residence area	Urban	61.7	38.3	5,075
		Rural	38.3	61.7	8,160
	Province of residence	Niassa	4.6	6.1	811
		Cabo Delgado	6.2	7.9	1,047
		Nampula	4.6	19.6	2,601
		Zambezia	4.1	18.0	2,381
		Tete	5.3	9.4	1,242
		Manica	6.3	7.0	924
		Sofala	9.0	8.4	1,108
		Inhambane	11.0	5.3	707
		Gaza	7.6	5.1	676
		Maputo Province	17.6	8.8	1,162
		Maputo City	23.6	4.4	579
	Total	percent	100.0	100.0	NA
		*n*	13,235		

### Participants’ COVID-19 vaccination status and vaccine acceptance and hesitancy

The study found that 77.2% [95% confidence interval (CI): 74.9–79.5] of participants reported receiving at least one dose of a COVID-19 vaccine. Among vaccinated women, 64.5% [95% CI: 62.2–66.7] reported receiving two doses. More than three-quarters [82.3, 95% CI: 79.1–85.1] of unvaccinated women reported that they would definitely take the vaccine if it was available and offered to them. An additional 5.8% [95% CI: 4.5–7.3] said they would likely take the vaccine if it was offered.

About 12% of unvaccinated respondents were hesitant to take the COVID-19 vaccine if offered (1.2% unlikely [95% CI: 0.8–1.8], 4.7% definitely not [95% CI: 3.5–6.3] and 6.0% undecided [95% CI: 4.1–8.7]). The primary reasons reported for not taking the vaccine were the dislike of needles (17.1% [95% CI: 12.4–23.0]), COVID-19 vaccine safety (12.0% [95% CI: 8.8–16.3]), COVID-19 vaccine effectiveness (10.2% [95% CI: 6.5–15.7]), medical reasons (5.6% [95% CI: 2.9–10.6]),waiting for others to get vaccinated first (5.4% [95% CI:2.5–11.1]), and eligibility age restrictions (3.2% [95% CI: 1.3–7.6]) (Table 2).

**Table 2:** Respondent COVID-19 vaccination characteristics.

Characteristics	Categories	Weighted percent	95% Confidence interval
Received dose of COVID-19 vaccine (*n* = 13,235)	Yes	77.2	[74.9–79.5]
	No	22.8	[20.5–25.1]
Number of doses received (*n* = 11,226)	1	35.5	[33.3–37.8]
	2	64.5	[62.2–66.7]
Likely to take vaccine if offered (*n* = 1974)	Yes, definitely	82.3	[79.1–85.1]
	Likely	5.8	[4.5–7.3]
	Unlikely	1.2	[0.8–1.8]
	No	4.7	[3.5–6.3]
	Undecided	6.0	[4.1–8.7]
Primary reason to not take vaccine (*n* = 557)	Not safe	12.0	[8.8–16.3]
	Needles	17.1	[12.4–23.0]
	Not effective	10.2	[6.5–15.7]
	Religious	1.6	[0.6–3.9]
	Wait for other first	5.4	[2.5–11.1]
	Various other reasons	38.1	[29.5–47.6]
	Medical reason	5.6	[2.9–10.6]
	Pregnant or breastfeeding	1.5	[0.9–2.4]
	Time	1.0	[0.5–1.9]
	Vaccine site challenges	1.0	[0.5–2.0]
	Fear	1.4	[0.4–4.8]
	Travel	1.1	[0.3–4.0]
	Age restrictions	3.2	[1.3–7.6]
	Local availability	0.9	[0.4–2.3]
	*n*	13,235	

Almost 90% [95% CI: 86.7–91.2] of women aged 40–49 years old had received the COVID-19 vaccine, compared to only 63.9% [95% CI: 57.2–70.0] of women under 20 years old (*p* < 0.001). Of the eleven provinces, women in Manica (89%), Gaza (88%), and Maputo City and Province (81%) had the highest vaccination rates (*p* = 0.003). About 80% [95% CI: 77.5–82.1] of women who had ever been pregnant or given birth had received the COVID-19 vaccine, compared to 63.9% [95% CI: 57.6–69.8] of women who had never been pregnant or given birth (*p* < 0.001). Place of delivery was associated with women’s COVID-19 vaccination rates, with 77.7% [95% CI: 73.5–81.4] of women who delivered at a health facility being vaccinated against COVID-19, compared to 65.2% [95% CI: 53.2–75.5] of those who delivered at home (*p* = 0.013) (Table 3).

**Table 3:** COVID-19 vaccination rates by sociodemographic and residential characteristics.

Characteristics	Categories	Received COVID-19 vaccine (%)	95% Confidence interval	*p*-value
Age group	<20	63.9	[57.2–70.0]	0.000
	20–29	76.0	[72.8–79.0]	
	30–39	84.1	[81.6–86.4]	
	40–49	89.1	[86.7–91.2]	
Education	None	77.4	[72.4–81.7]	0.966
	Primary	77.0	[73.5–80.2]	
	Secondary and above	77.5	[74.7–80.1]	
Marital status	Married or cohabitating	78.3	[75.7–80.8]	0.000
	Widowed, divorced, or separated	86.9	[82.8–90.1]	
	Never married	66.4	[61.1–71.4]	
Residence area	Urban	79.8	[76.3–82.9]	0.077
	Rural	75.7	[72.4–78.7]	
Province of residence	Niassa	79.0	[70.0–85.9]	0.003
	Cabo Delgado	73.8	[66.4–80.1]	
	Nampula	77.6	[70.8–83.2]	
	Zambezia	70.3	[62.6–77.1]	
	Tete	72.5	[63.9–79.8]	
	Manica	88.5	[84.7–91.5]	
	Sofala	75.7	[65.6–83.5]	
	Inhambane	79.1	[74.5–83.0]	
	Gaza	88.4	[83.5–91.9]	
	Maputo Province	80.8	[77.8–83.5]	
	Maputo City	80.6	[77.5–83.4]	
Ever pregnant or given birth	Yes	79.9	[77.5–82.1]	0.000
	No	63.9	[57.6–69.8]	
Currently pregnant (*n* = 11,636)	Yes	78.4	[71.9–83.8]	0.527
	No	80.2	[77.9–82.3]	
ANC services (*n* = 3,185)	Yes	75.9	[71.4–79.9]	0.932
	No	74.9	[47.4–90.8]	
	<4	77.0	[72.6–80.9]	0.207
	4≥	72.2	[63.4–79.6]	
Place of delivery (*n* = 3,185)	Home	65.2	[53.2–75.5]	0.013
	Health facility	77.7	[73.5–81.4]	
Mobile phone ownership	Respondent	80.7	[77.9–83.2]	0.000
	Husband or shared	78.2	[74.7–81.3]	
	Other	65.4	[59.7–70.7]	
Need permission to use phone (*n* = 8,415)	Yes	82.9	[76.4–87.9]	0.450
	No	80.3	[77.2–83.1]	
Can access phone when wanted	Yes	78.6	[76.0–81.0]	0.042
	No	74.2	[70.1–78.0]	
Makes decisions about respondent’s health	Respondent	81.9	[78.9–84.5]	0.000
	Husband or shared	77.3	[74.1–80.1]	
	Other	67.2	[62.0–72.0]	
Makes decisions about child(ren)'s health	Respondent	77.1	[73.9–80.0]	0.249
	Husband or shared	78.1	[74.9–81.0]	
	Other	70.9	[61.5–78.7]	
	*n*	13,235		

Characteristics relating to phone ownership and health decision-making were also associated with respondents’ COVID-19 vaccination status. About 81% [95% CI: 77.9–83.2] of respondents who owned the phone they were speaking on had received the COVID-19 vaccine compared to 65.4% [95% CI: 59.7–70.7] of those who reported that someone else owned the mobile phone (*p* < 0.001). About 82% [95% CI: 78.9–84.5] of women who reported that they usually make decisions about their own healthcare had received the COVID-19 vaccine compared to 67.2% [95% CI: 62.0–72.0] of women who reported that someone else makes decisions about their healthcare (*p* < 0.001).

### Factors associated with the likelihood of receiving the COVID-19 vaccine

Controlling for other factors, women aged 20 years and above were more likely to receive the COVID-19 vaccine than women under 20 years of age (for 40–49-year-old age group: adjusted odds ratio [aOR] = 3.57; 95% CI: 2.27–5.62). Educated women were more likely to receive the COVID-19 vaccine than uneducated women (for secondary level and higher: aOR = 1.67; 95% CI: 1.17–2.37). Women who had ever been pregnant or given birth were more likely to receive the COVID-19 vaccine than women who had never been pregnant or given birth (aOR = 1.50; 95% CI: 1.01–2.23). Women who reported giving birth at a health facility were more likely to receive the COVID-19 vaccine than women who gave birth at home (aOR = 1.69; 95% CI: 1.02–2.79). Women in nearly all provinces were more likely to receive the COVID-19 vaccine than women in Cabo Delgado (for Manica: aOR = 2.67 (95% CI: 1.60–4.44); for Gaza: aOR = 2.84 (95% CI: 1.59–5.08)).

However, respondent characteristics such as marital status, place of residence, and women’s empowerment based on mobile phone ownership, permission and access to use the phone, and decision-making about health care were not significantly associated with vaccination status in the multivariate analysis (Table 4).

**Table 4:** Regression results on women’s COVID-19 vaccination status.

Characteristics	Categories	Bivariate		Multivariate		
		OR	95% Confidence interval	aOR	95% Confidence interval	
Age group	<20	Ref		Ref		
	20–29	1.79***	(1.31–2.44)	1.50**	1.07	2.12
	30–39	2.99***	(2.20–4.07)	2.35***	1.62	3.42
	40–49	4.64***	(3.22–6.69)	3.57***	2.27	5.62
Education	None	Ref		Ref		
	Primary	0.98	(0.71–1.35)	1.21	0.85	1.72
	Secondary and above	1.01	(0.76–1.34)	1.67***	1.17	2.37
Marital status	Married or cohabitating	Ref		Ref		
	Widowed, divorced, or separated	1.84***	(1.31–2.58)	1.30	0.87	1.95
	Never married	0.55***	(0.42–0.70)	0.85	0.59	1.22
Residence area	Rural	Ref		Ref		
	Urban	1.27*	(0.97–1.65)	1.09	0.82	1.45
Province of residence	Cabo Delgado	Ref		Ref		
	Niassa	1.33	(0.73–2.42)	1.30	0.74	2.27
	Nampula	1.23	(0.74–2.03)	1.18	0.71	1.96
	Zambezia	0.84	(0.51–1.39)	0.82	0.48	1.41
	Tete	0.94	(0.55–1.60)	0.86	0.49	1.49
	Manica	2.74***	(1.68–4.46)	2.67***	1.60	4.44
	Sofala	1.10	(0.60–2.01)	1.09	0.59	2.02
	Inhambane	1.34	(0.86–2.08)	1.04	0.66	1.66
	Gaza	2.69***	(1.57–4.61)	2.84***	1.59	5.08
	Maputo Province	1.49*	(1.00–2.22)	1.12	0.73	1.72
	Maputo City	1.47*	(0.98–2.20)	1.17	0.74	1.87
Ever pregnant or given birth	No	Ref		Ref		
	Yes	2.25***	(1.68–3.01)	1.50**	1.01	2.23
Currently pregnant	Yes	Ref				
	No	1.11	(0.80–1.55)			
ANC services	No	Ref				
	Yes	1.05	(0.31–3.54)			
	<4	Ref				
	4≥	1.29	(0.87–1.91)			
Place of delivery	Home	Ref		Ref		
	Health facility	1.86**	(1.14–3.04)	1.69**	1.02	2.79
Mobile phone ownership	Husband or shared	Ref		Ref		
	Respondent	1.17	(0.93–1.47)	0.85	0.62	1.16
	Other	0.53***	(0.40–0.70)	0.60***	0.42	0.86
Need permission to use phone	Yes	Ref				
	No	0.84	(0.54–1.31)			
Can access phone when wanted	No	Ref		Ref		
	Yes	1.28**	(1.01–1.61)	0.89	0.65	1.21
Makes decisions about respondent’s health	Husband or shared	Ref		Ref		
	Respondent	1.33**	(1.07–1.66)	1.16	0.85	1.59
	Other	0.60***	(0.46–0.80)	0.78	0.53	1.15
Makes decisions about child(ren)'s health	Husband or shared	Ref		Ref		
	Respondent	0.95	(0.74–1.20)	1.32*	0.96	1.81
	Other	0.68*	(0.45–1.03)	0.94	0.59	1.52

*** *p* < 0.01, ** *p* < 0.05, * *p* < 0.1.

*n* = 13,235.

## Discussion

The RaMMPS study, conducted between March–July 2022, demonstrated that mobile phone surveys are a feasible approach to quickly collect data to assess the impact of COVID-19 pandemic on vaccine uptake among women of reproductive age in Mozambique. COVID-19 vaccination coverage was high (77.2%) among studied women ages 15–49. This estimate was slightly lower than findings from a national study, which reported a vaccination coverage of 93% among adults over 18 years as of May 2022 (41). Additionally, among vaccinated women in the RaMMPS study, 64.5% reported receiving two doses. The COVID-19 vaccination rate of the general population, which was defined as the “total number of people who received all doses prescribed by the initial vaccination protocol, divided by the total population of Mozambique,” was estimated at 46% as of May 2022 (41). These findings suggest that overall the vaccination coverage in Mozambique is relatively high compared to many other sub-Saharan African countries (12).

Among unvaccinated women in the RaMMPS study, 88% reported being likely to take the COVID-19 vaccine if it was offered to them. This finding was higher compared to a 2021 study in Mozambique that revealed that 71% of participants indicated they would take the vaccine, with significant sociodemographic differences across subpopulations (8). This reflects a high level of vaccine acceptance, especially given that women are generally more likely to be vaccine-hesitant than men (1, 7, 20, 22, 24). Concerns about vaccine safety and effectiveness were among the primary reasons for reluctance among women unwilling to be vaccinated. Similar concerns have been reported in other studies in sub-Saharan Africa, including in Uganda, Zimbabwe, Tanzania, Ethiopia, Nigeria, Uganda, Malawi and Burkina Faso (1, 15, 16, 20, 24).

We found that the odds of COVID-19 vaccination increased with age, with women aged 40–49 being significantly more likely to be vaccinated compared to those under 20. Our findings were consistent with previous studies conducted in Mozambique (8, 23, 26). Similar age-related patterns in COVID-19 vaccine acceptance have been also observed in studies from Botswana, Zambia, Zimbabwe, Tanzania and the Democratic Republic of Congo (7, 24, 42–44). However, a multi-country study showed that younger age was associated with vaccine acceptance (20). When analyzing these associations, it is important to consider that the COVID-19 vaccination strategy in Mozambique has prioritized older age groups early in the rollout (11), which may have influenced both access and uptake among different age cohorts.

We found that women who had attained secondary education or higher were more likely to be vaccinated than women without formal education. However, there was no significant difference in COVID-19 vaccination coverage between women with only primary education and those with no formal education. The relationship between education level and vaccine acceptance has varied across studies. In some countries, including Botswana, Burkina Faso, Ethiopia, Malawi, Nigeria, Sierra Leone, and Uganda, with lower levels of formal education were associated with higher vaccine acceptance (15, 42). Conversely, other countries including Senegal, Zimbabwe and Kenya have found that higher levels of education was associated with increased acceptance of COVID-19 vaccine (16, 19, 45, 46).

Women’s health behavioral factors related to the use of health facilities during pregnancy increased the likelihood of being vaccinated against COVID-19. Specially, women who delivered at a health facility were significantly more likely to be vaccinated against COVID-19 than those who delivered at home. However, ANC attendance was not significantly associated with COVID-19 vaccine acceptance, likely due to the near-universal ANC coverage observed in our study. This may also be related to Mozambique’s COVID-19 vaccination policy, which excluded pregnant women and individuals under 18 years of age from initial eligibility (26). While some studies have examined COVID-19 vaccine acceptance among pregnant women, including one in Ethiopia that assessed factors influencing uptake among ANC clinic attendees, few have explored the association between birth history factors and COVID-19 vaccine acceptance (47).

Geographic variation in vaccine acceptance was also observed. Women residing in provinces such as Manica and Gaza reported significantly higher levels of COVID-19 vaccine acceptance than women in Cabo Delgado. This may be related to the military conflicts in Cabo Delgado that have led to population displacement and disruption of community and health facilities services (48). Similarly, findings from another 2022 study conducted in the COMSA platform indicated that 65% of women 15–49-year-olds in Nampula and 80% in Maputo (Province and City) were vaccinated against COVID-19 (49). Our findings were also consistent with an early study conducted in Mozambique between September 2020 and March 2021 that revealed that COVID-19 vaccine acceptancy was not significantly associated with women’s place of residence (26). However, evidence from other sub-Saharan African countries is mixed. A multi-country phone survey study reported higher vaccine acceptance in rural areas of Burkina Faso, Ethiopia and Malawi (15), of Madagascar (50) and Bangladesh (6), while other studies have identified rural residence as a factor associated with increased vaccine hesitancy (5, 6, 51). In Kenya, findings from phone-based surveys varied across different rounds of data collection, showing inconsistent patterns in vaccine acceptance between urban and rural populations (45). Accessibility of healthcare infrastructure and vaccination services emerged as key determinants of increased COVID-19 vaccine uptake (6, 15, 50).

Characteristics related to women’s empowerment- measured through mobile phone ownership, access, and participation in healthcare decision-making- were not significantly associated with COVID-19 vaccination in the multivariate regression model. To our knowledge, no prior studies have specially examined the relationship between women’s empowerment and COVID-19 vaccine acceptance in Mozambique or other LMIC settings.

Vaccine hesitancy is influenced by a complex interplay of sociocultural, political, informational, and logistical factors. Trust in health systems, individual knowledge, perceived risks and benefits, cultural norms, and healthcare access can all affect vaccination decisions (1, 8, 19, 52, 53). Our study identified factors linked to higher vaccine uptake such as age group, education, marital status, geographic location, and maternal healthcare use. These insights can guide targeted strategies to that address specific demographic, social geographic characteristics to enhance vaccine acceptance. For future public health emergencies, understanding these drivers of vaccine behavior is critical to designing effective disease prevention campaigns. By strengthening trust, improving access, and targeting communication efforts, health systems can improve immunization rates and better manage outbreaks through inclusive and responsive public health planning.

### Study limitations

Our study has several important limitations. First, the study used only a sample of women and households with phones. Therefore, many women without phones were excluded from the study, which might impact its representativeness. However, we employed the post-stratification weighting to reduce this bias (35, 38). Second, the coverage and non-response biases are common in mobile phone surveys, and participation in this study was limited to women ages 15–49 with access to a mobile phone (54). Studies suggest that people with lower levels of education, in rural areas, and of lower income groups are less likely to have mobile phones, which can lead to coverage bias in mobile phone surveys (55, 56). Additionally, there may be incomplete information on sampled individuals if the survey ends early, or if there are differences between respondents and non-respondents, which creates non-response bias (56). In addition, social desirability bias could have impacted participants’ responses, and subsequently the study findings.

## Conclusion

This study contributes to Mozambique-specific evidence on factors influencing COVID-19 vaccine acceptance and hesitancy among women, while examining other potential factors of influence, including women’s empowerment. It highlights the importance of targeting younger women, those with no formal educated, women delivering outside a health facility, and those residing in conflict-affected provinces, like Cabo Delgado. While ANC attendance was not significantly associated with COVID-19 vaccine acceptance, improving the quality of counseling during ANC services – specially around vaccine safety and effectiveness is critical. However, our findings are limited by potential biases related to phone ownership and social desirability affecting respondents’ willingness to report their vaccination status and factors related to vaccine hesitancy. Further research, including qualitative studies, is recommended to better understand women’s decision-making and strengthen vaccines uptake strategies.

## Data Availability

The raw data supporting the conclusions of this article will be made available by the authors, without undue reservation.
